# Evaluation of Peri-Implant Parameters and Functional Outcome of Immediately Placed and Loaded Mandibular Overdentures: A 5-year Follow-up Study

**DOI:** 10.3290/j.ohpd.b4836045

**Published:** 2024-01-15

**Authors:** Abdulaziz A. AlHelal, Abdulaziz A. Alzaid, Saad H. Almujel, Mohammed Alsaloum, Khalid K. Alanazi, Ramzi O. Althubaitiy, Khulud A. Al-Aali

**Affiliations:** a Associate Professor, Department of Prosthetic Dental Sciences, College of Dentistry, King Saud University, Riyadh, Saudi Arabia. Study design, treated patients, methodology, investigation, manuscript writing.; b Assistant Professor, Restorative and Prosthetic Dental Science Department, College of Dentistry, King Saud Bin Abdulaziz University for Health Sciences, Riyadh, Saudi Arabia; King Abdullah International Medical Research Center, Riyadh, Saudi Arabia. Conceptualisation, software, project management, manuscript review, investigation.; c Assistant Professor, Department of Prosthetic Dental Sciences, College of Dentistry, King Saud University, Riyadh, Saudi Arabia. Conceptualisation, software, project management, manuscript review, investigation.; d Assistant Professor, Restorative and Prosthetic Dental Science Department, College of Dentistry, King Saud Bin Abdulaziz University for Health Sciences, Riyadh, Saudi Arabia; King Abdullah International Medical Research Center, Riyadh, Saudi Arabia. Conceptualisation, software, project management, manuscript review, investigation.; e Assistant Professor, Conservative Dental Science Department, College of Dentistry, Prince Sattam Bin Abdulaziz University, Alkharj 11942, Saudi Arabia. Conceptualisation, software, project management, manuscript review, investigation.; f Assistant Professor, Department of Prosthodontics, College of Dentistry, Prince Sattam bin Abdulaziz University, Alkharj 11942, Saudi Arabia. Conceptualisation, software, project management, manuscript review, investigation.; g Associate Professor, Department of Clinical Dental Sciences, College of Dentistry, Princess Nourah Bint Abdulrahman University, Riyadh, Saudi Arabia. Conceptualisation, software, project management, manuscript writing, funding, investigation.

**Keywords:** dental implants, immediate dental implant loading, overdentures, peri-implantitis

## Abstract

**Purpose:**

To evaluate the peri-implant parameters of immediately placed and loaded mandibular overdentures over a 5-year follow-up period.

**Materials and Methods:**

All subjects who had been advised and planned for two-implant mandibular overdenture treatment were included in this study. The peri-implant parameters –including plaque index (PI), bleeding index (BI) and peri-implant pocket depth (PIPD) as well as marginal bone loss (MBL) – were assessed. In addition, prosthodontic parameters including abutment-, implant- and denture-related complications were assessed. Patients were evaluated at follow-up visits, scheduled at 1, 12, 24, 36, 48, and 60months. The data distribution was analysed with the Shapiro-Wilk test. Data within follow-up categories were compared using ANOVA and the Tukey-Kramer test. A p-value <0.05 was considered statistically significant.

**Results:**

Among the 32participants, 19 were males and 13 were females, with a mean age of 60.5±7.33. The mean plaque index (PI), bleeding index (BI) and peri-implant pocket depth (PIPD) varied over time. However, no statistically significant difference was observed in the plaque index, bleeding index and peri-implant pocket depth over time (p>0.05). The mean value at baseline was found to be -0.9±0.3. The values increased over time, with the highest value observed at 60months 2.6±0.7, which was statistically significant (p<0.001).

**Conclusion:**

Immediately placed and loaded mandibular implant overdentures using two un-splinted implants with locator attachments showed acceptable PI, BI and PIPD at the 5-year follow-up. Statistically significantly greater marginal bone loss was observed from baseline to follow-up, but it was within acceptable limits. A moderate number of restorative and abutment complications were observed during the follow-up of IODs.

Edentulism, meaning the state of having no teeth, is a chronic condition.^1^ From a prosthodontic perspective, it leads to adverse aesthetic and biomechanical sequelae, including residual ridge resorption, degenerative changes, impaired masticatory function and loss of neuromuscular control.^1^^,^^21^ However, loss of teeth and associated tissues also compromises the quality of life of an individual. Conventionally, removable complete dentures are used to restore the oral function and appearance of an edentulous patient. Conventional complete dentures (CCD), particularly in the mandibular arch, possess limited stability and retention due to a reduced denture-bearing area and tongue. As a result, CCD are associated with ill-fitting dentures, denture trauma, pain, loss of patient confidence, and functional and phonetic deficiencies. Moreover, continued bone loss reduces the success of future management strategies. With the advent of dental implants, the functional deficiencies associated with CCD have been greatly ameliorated.^23^^,^^22^^,^^29^

Implant dentistry in the modern era extends far beyond simplistic implant surgery and restoration; it now entails a comprehensive and refined approach. Implant-supported overdentures (IODs) have emerged as a highly successful treatment modality to rehabilitate completely edentulous patients. The success rate of IODs is contingent upon multiple factors, such as meticulous patient selection, strategic implant placement, potential need for periodontal therapy, overall patient health, bone quality, surgical and prosthetic techniques, and maintenance. Undoubtedly, clinicians also play a pivotal role in securing long-term success.^4^^,^^24^^,^^25^ Moreover, IODs offer many advantages over CCD; they not only improve oral function by increasing the retention and stability of the prosthesis but also preserve the residual bone and improve the quality of life of edentulous patients.^5^^,^^14^ Misch^18^ proposed five organised treatment options for overdentures (ODs) for the mandibular arch. They range from primarily soft-tissue support and implant retention to a completely implant-supported prosthesis with rigid stability. It is proposed that the prostheses could be supported by two to five anterior implants placed in planned, specific sites.^18^ Even so, the number of implants can also be determined by the consensus between the restoring dentist and the patient.^11^ The minimum number of implants needed for implant restoration is still debatable. The McGill and York consensus is strongly in favour of the two-implant–supported overdenture (TISOD).^8^^,^^9^

The literature suggests that for IODs, the number, diameters, surface topography, and attachment system play an important role.^8^^,^^9^^,^^27^^,^^31^^,^^35^^,^^36^ Conventionally, it has been suggested that four splinted implants are required to achieve a long-term satisfactory outcome for an immediately placed and loaded IOD.^36^ However, various studies showed promising results with 2 or 3 immediately placed and loaded IODs.^27^^,^^31^^,^^35^ Even immediate loading of one single implant has been suggested.^16^ The immediate placement and loading of the implant overdentures allow a simplified procedure, shorter treatment duration, and a stable prosthesis; frequent relining of transitional prostheses during the healing period is avoided and oral function is immediately restored. Conversely, mechanical stress exerted on implants during the healing period may result in micro-motions at the implant/bone interface, thus interfering with the healing process.^27^^,^^36^ Despite this, the literature reports that the immediate loading of implants shows high success rates in the mandible, regardless of the type of attachment systems used, surface characteristics and splinting.^2^

Different types of attachment systems have been proposed for IODs. Both splinted and non-splinted attachments have their own indications and requirements.^15^ However, non-splinted attachments have gained popularity due to smaller space requirements, ease of cleaning, cost-effectiveness and lower technique sensitivity. Among the non-splinted attachments, the locator attachment has become popular as it offers dual retention, which increases the stability of the IOD along with a higher retentive force, ensuring a secure fit of IOD.^15^

Various clinical studies have reported immediately placed and loaded IODs to be an acceptable and successful treatment modality for edentulous mandibles.^2^^,^^15^^,^^31^^,^^35^ But most of these studies were limited to a period of 1 to 2years, hence failing to evaluate the peri-implant parameters over an extended period to determine the long-term outcomes. We hypothesised that immediately placed and loaded mandibular overdentures will demonstrate favourable peri-implant parameters over a 5-year follow-up period, including bone loss, healthy peri-implant soft tissues and satisfactory functional outcomes. Understanding the peri-implant parameters associated with immediate loading in the context of IODs is highly clinically relevant, as it directly impacts patient care and influences treatment planning decisions. To provide evidence-based guidance to clinicians and patients, it is essential to conduct a thorough assessment of the long-term outcomes of this treatment approach. Unfortunately, previous studies in this area have been limited to relatively short follow-up periods of 1-2years.^31^^,^^35^ As a result, they have not been able to fully evaluate the peri-implant parameters over an extended period to determine the treatment’s true long-term effectiveness. Therefore, this study was conducted with the aim to evaluate the peri-implant prosthetic parameters and functional outcome of immediately placed and loaded mandibular overdentures over a 5-year follow-up period to address this gap in knowledge.

## MATERIALS AND METHODS

The patients were recruited and the procedures performed at a specialist dental practice. The study protocol was reviewed and prior ethical approval was given (SPRC-033-018). The ethical standards were in accordance with the declaration of Helsinki and its modifications (2013). The study protocol, risks and benefits, other treatment options and finances were discussed with the patient before written consent was obtained. Participants were free to opt out of the study at any time without consequences.

**Fig 1:**
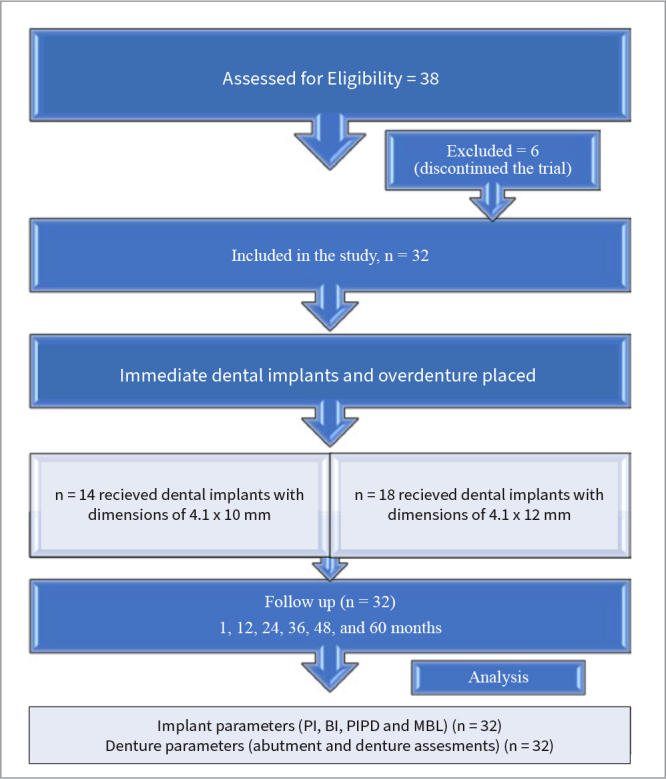
CONSORT flow diagram.

All patients included were consulted and indicated for two-implant mandibular overdenture treatment. Patients with jaw-related abnormalities, periodontal disease in last 12months, general health diseases including but not limited to uncontrolled diabetes, cancer or chemotherapy, bone disease, pregnancy or osteoporosis, diseases influencing bone metabolism, immunocompromised disease and conditions contraindicating oral surgery were excluded. Participants with complaints and compromised existing dentures were included. The patient selection protocol is presented in Fig1.

**Fig 2:**
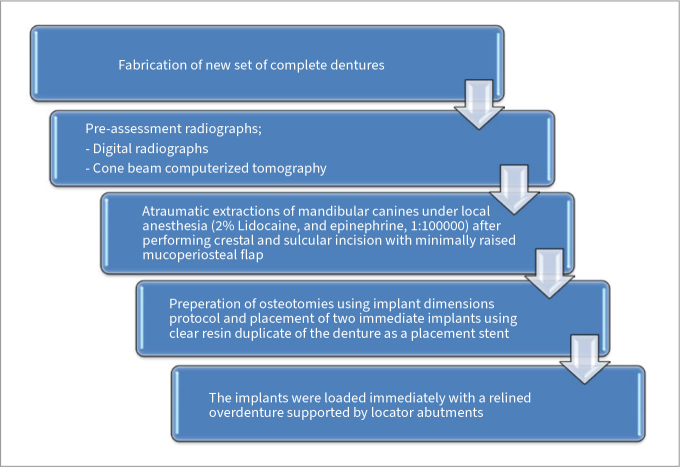
Implant placement and loading protocol.

The implant placement protocol is explained in Fig2. Implants with 4.1 x 10 or 4.1 x 12mm (Roxolid implant- ITI, Straumann; Basel, Switzerland) were immediately placed in the study participants. A board-certified, surgically trained prosthodontist placed the implants. Surgical closure was performed with interrupted 3-0 Vicryl (Ethicon; Cincinnati, OH, USA) sutures. A post-operative digital radiograph (Planmeca; Helsinki, Finland) was performed to evaluate implant location, position and bone levels.

Locator abutments were placed and torqued to 15Ncm, and space was created within the fitting surface of dentures having no contact with the locator housing. A locator metal housing with retentive caps was secured to the abutments and checked to ensure they were at the same occlusal height. The reline pick-up was performed in occlusion with opposing dentures using self-curing hard-denture reline (UFI Gel, PMMA, Voco; Cuxhaven, Germany). The implant overdenture prosthesis was removed, finished and polished in the laboratory. All prosthetic procedures were performed by two board-certified and trained prosthodontists.

The post-operative care prescribed included the use of analgesics (Ibuprofen, 600mg, 8h), antibiotics (amoxicillin, 500mg, every 8h for 5days), gentle oral rinsing and cleaning of the denture with 0.12% chlorhexidine for 10days. Patients were recalled in 2weeks.

**Fig 3:**
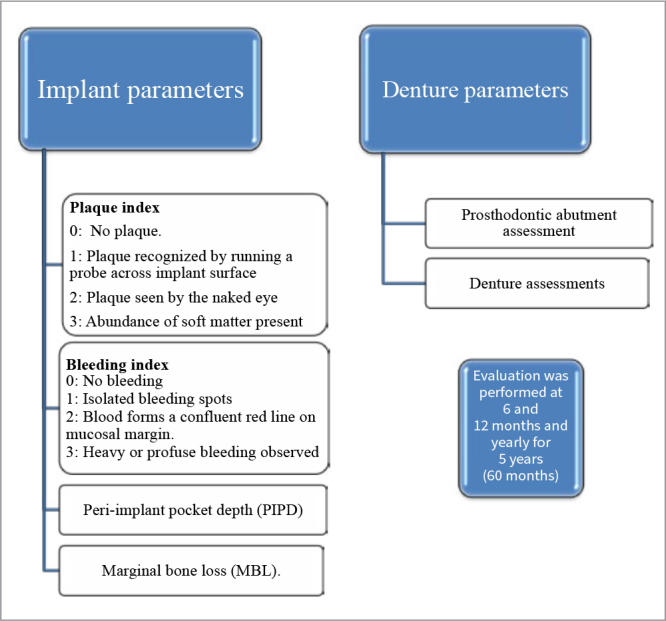
**Fig 3** Evaluation of peri-implant parameters at follow-up visits.

Implants and denture parameters were evaluated using certain indices, as shown in Fig3. For all evaluations, means and standard deviations were calculated. Two examiners performed evaluations, and inter-examiner training and reliability (kappa scores) were assessed. Evaluations were performed by running the periodontal probe around the implant parallel to the abutment surfaces. Modified forms of PI and BI suggested by Mombelli et al^20^ were used. The distance between the gingival margin and the most apical probable portion was recorded in mm using a manual pressure-sensitive probe (CP12, Hu-Friedy; Chicago, IL, US) with a slight force of about 0.2N at 6 aspects for each implant (mesio-buccal, mid-buccal, disto-buccal, mesio-lingual, mid-lingual, disto-lingual).^28^ Marginal bone loss (MBL) was assessed using digital intraoral periapical radiographs with a paralleling technique and an intraoral stent for standardised positioning. Bone levels were evaluated after calibration using Romexis Software (Promax 3D Classic; Helsinki, Finland). Bone levels were evaluated from implant abutment junction to radiographic mesial and distal bone crest to implant.^28^

Evaluation of denture parameters included prosthodontic abutment assessment and denture assessments. The number of complications was observed and reported at 12, 24, 36, 48 and 60months of follow-up. The parameters included loose abutment, abutment fracture, implant fracture, abutment corrosion, loose retentive cap, loss of retentive cap, replacement of retentive female, loose metal housing and loss, denture fracture, rebase, denture rebase and denture replacement. The peri-implant and restorative parameter evaluations were performed by a board-certified prosthodontist. The inter-examiner reliability was assessed, yielding a kappa score of 0.88.

The data were tested for normal distribution with the Shapiro-Wilk test (SPSS, Version 21, IBM; Armonk, NY, USA). The biological data comprising PI, BI, PIPD and MBL were compiled as means and standard deviations (SD). Data were compared within follow-up categories using ANOVA and the Tukey-Kramer test. Prosthodontic parameters were analysed using descriptive analysis. p≤0.05 was considered statistically significant.

## RESULTS

A total of 38 patients were initially included in the trial. However, two patients died and four patients discontinued the trial due to relocation, leaving a total of 32 patients. Thirty-two patients were assessed for all evaluations and parameters throughout the study, as presented in Fig1. Of a total of 32 participants, 19 were males and 13 were females, with a mean age of 60.5±7.33. The mean service time of the removable partial denture was 68.5±23.5months (Table 1).

**Table 1:** General characteristics of patients included (n=32)

Variables	Mean	SD
Age	60.5	7.33
Gender (M/F)	19/13	
Time in service of removable partial denture (months)	68.5	23.5

The peri-implant soft tissue parameters including PI, BI and PIPD were assessed at different time intervals (baseline and 12, 24, 36, 48, and 60months). The mean PI score at baseline was 1.08±0.4; it varied at different time intervals but did not show any statistically significant pattern. The highest mean PI score was observed at 60months (1.70±0.7). However, no statistically significant differences were observed in PI over time (p>0.05).

The mean BI score at baseline was 1.04±0.6. The mean BI scores also fluctuated throughout the study. Initially, it tended to decrease, with the lowest mean BI score of 0.80±0.5 observed at 24months. Later, an increasing tendency was observed as of 36months, and the highest mean BI score was observed at 60months (1.34±0.7). However, no statistically significant difference was observed over time (p>0.05).

The mean values for PIPD also showed some variation over time. The PIPD mean score at baseline was 3.50±1.0, which fluctuated over different periods of time. A marked increase in the mean score (4.10±1.6) was observed at 48months, which remained constant up to the 60th month. Here too, no statistically significant difference was observed over time (p>0.05; Table 2).

**Table 2:** Peri-implant soft tissue parameters from baseline (control) to 5years function (means, SD and p-value)

Soft tissue parameter	Baseline (1month)	12months	24months	36months	48months	60months	Overall p-value
PI	1.08±0.4	1.7±0.5	0.90±0.5	1.30±0.6	1.53±0.5	1.70±0.7	>0.05
BI	1.04±0.6	0.85±0.5	0.80±0.5	1.20±0.6	1.30±0.7	1.34±0.7	>0.05
PIPD	3.50±1.0	3.42±1.1	3.50±1.2	3.40±1.1	4.10±1.6	4.40±1.2	>0.05

PI: plaque index; BI: bleeding index; PIPD: peri-implant pocket depth.

Marginal bone loss (MBL) was also measured at different time intervals (baseline and 12, 24, 36, 48, and 60months). The mean value at baseline was -0.9±0.3. The values increased over time, with the highest value observed at 60months 2.6±0.7, which was statistically significant (p<0.001), as presented in Table 3.

**Table 3:** Peri-implant bone levels from baseline (control) to 5years function (means, SD and p-value)

Clinical parameter	Baseline (1month)	12months	24months	36months	48months	60months	Overall p-value*
MBL	-0.9±0.3 a	0.70±0.3 b	1.7±0.5 c	1.90±0.4 c	2.4±0.5 d	2.6±0.7 d	<0.001

MBL: marginal bone loss; *ANOVA.

Various prosthetic complications were observed over a period of 5years. As far as abutment-related complications are concerned, abutment loosening was observed in 26 implants, followed by wear observed in 12 abutments and occlusal adjustments in 27 cases. No case of abutment or implant fracture was reported. Moreover, certain complications related to retention elements were also observed at follow-up visits. In 15 cases, the metal housing used for retention became loose. The retentive female component was broken in 28 cases and loose in 26 cases. Replacement of the retentive male component was required in 26 cases.

Lastly, complications associated with the IODs were also noted; relining was required in 25 cases, followed by rebasing in 7 cases. Fracture of IOD was observed in 6 cases and new dentures replaced 6 IODs, as presented in Table 4.

**Table 4:** Prosthetic complication frequency among IODs over 5years

Complication types	Frequency (n=64 implants)
Abutment (A)	
A. Loosening	26
A. Wear	12
A. Fracture	-
Implant fracture	
Occlusal adjustment	27
Retention element	
Metal housing loose	15
Retentive female broken	28
Retentive female loose	26
Replacement of retentive male	26
Denture	Frequency (n = 32 IOD)
Reline	25
Rebase	7
Fracture	6
IOD replacement	6

## DISCUSSION

The immediate placement and loading of mandibular overdentures present a distinctive approach to dental implant treatment, enabling the immediate restoration of edentulous mandibular arches. The purpose of this study was to evaluate the peri-implant parameters of immediately placed and loaded mandibular IODs over a 60-month follow-up. The extended observation period enabled a comprehensive evaluation of the implants and peri-implant tissues over a substantial period. The hypothesis was partially accepted, as immediately placed and loaded TISOD demonstrated acceptable soft and hard tissue parameters, including PI, BI, PIPD and MBL.

In the present study, two non-splinted implants of standard dimensions (4.1 x 10 or 4.1 x 12mm) were placed immediately after extraction of mandibular canines to support an immediately loaded overdenture (IL-OD). This is in accordance with other studies in which two standard-sized implants were used to support mandibular IL-OD,^6^^,^^10^^,^^17^^,^^31^ while other researchers used four standard-sized implants. Initially, when the immediate-implant protocol for IL-OD first began to be implemented, four implants were considered mandatory to optimise the biomechanical load distribution. However, there were no long-term studies to support this. Since then, however, other published data have demonstrated that TISOD showed similar survival/success rates or clinical outcomes, albeit with follow-up periods of only 1-3years. An exception is one retrospective study, which demonstrated a survival rate of 95.5% after 20years of loading, thus suggesting TISOD to be a very reliable therapy for patients with an edentulous mandible.^34^

In this study, 2 non-splinted implants were used with locator abutments. In a systematic review and meta-analysis on IOD attachments, Chaware et al^3^ found the survival rate of attachments to be in the range of 95%–97% for bar attachments, 96%–100% for ball, 90%–92% for magnet, and 97% for locator attachments after a mean follow-up period of 3years. They also reported that the bar attachments were associated with moderate a peri-implant tissue response, including mucosal inflammation as a result of plaque and calculus accumulation, in addition to bone loss.^7^ It is suggested that locator attachments require more frequent maintenance and repairs; however, magnetic attachments resulted in higher levels of bone loss and were prone to displacement during functional activities.^3^^,^^7^ In addition, in terms of patient satisfaction with the use of IOD, it was higher with ball, locator, and bar attachments, while satisfaction was low for magnetic attachments. Therefore, locator attachments have demonstrated excellent performance in terms of survival rate, tissue response and patient satisfaction, and are recommended for IODs.^3^

In the present study, the peri-implant soft tissue parameters including PI, BI and PIPD were assessed at different time intervals up to 60months. The mean PI, BI and PIPD scores fluctuated throughout the study. However, no statistically significant difference was observed over time (p>0.05). This is in accordance with the study by Elsyad et al,^7^ which showed no statistically significant differences in the majority of the clinical and radiographic parameters for two implant attachment IOD systems. With the use of locator attachments, no statistically significant difference was observed over time with the PI and BI scores. However, in contrast to the present study, PIPD increased statistically significantly with time, and a statistically significant difference was observed over time (p<0.01). Those authors^7^ believed that the increased PIPD might be due to increased vertical bone loss observed with time. In a similar study,^6^ clinical parameters of the conventional, immediately loaded TISOD with ball-retained attachments were compared. PIPD at distal and labial sites in the immediate loading group were higher than the conventional loading group, while BI and PI showed no statistically significant differences between the two groups. Increased PIPD was a result of increased bone loss, while the low PIPD score at a later stage was due to the following gingival recession. Salman et al^26^ conducted a 5-year follow-up study to compare immediate vs delayed loading of mandibular IOD supported by 2 non-splinted locator attachments, and found similar clinical outcomes among the groups.

In the present study, MBL observed at 1year of loading was 0.70±0.3, which is similar to other studies including those of Marzola et al,^17^ Stricker et al^32^ and Turkyilmaz et al^33^ (0.3 to 0.7mm) after 1 year. However, in this study, the MBL increased statistically significantly (p<0.01) over time, with the highest value observed at 60months 2.6±0.7. Elsyad et al^6^ found that vertical bone loss (VBL) and horizontal bone loss (HBLO) increased significantly after 3years (p=0.03) in the IL-OD group, while in the delayed loading group, VBL and HBLO did not change statistically significantly over time. According to Misch et al^19^ and Heckmann et al,^12^ the VBL can be attributed to two factors: a decreased area of bone support and increased movement of the prosthesis supported by non-splinted implants. The increased movement of the prosthesis results in higher forces acting on the implants, which consequently amplifies the bending moment and contributes to VBL. The authors suggested splinting the implants together to improve biomechanical force distribution. As in this study, two non-splinted implants placed in the canine region have the potential to create bending moments around the implants by acting as a fulcrum, resulting in increased bone loss. Conversely, other studies found no statistically significant differences in the MBL between immediate- and conventional-loading protocols over a period of 1year.^17^^,^^33^^,^^36^

Various complications were observed in the present study, including abutment loosening, wear and occlusal adjustments, loose metal housing and loose/broken female/male components. Similar complications were reported by Kutkut et al,^14^ who observed that some subjects required frequent follow-up visits for the replacement of damaged locator attachments/locator abutments. Stoker et al^31^ reported about prosthesis maintenance only in the 15 cases that underwent relining/repair. In the present study, relining was necessary in 25 cases, followed by rebasing in 7 cases, and 6 IODs experienced fractures. In contrast, Kronstrom et al^13^ reported low maintenance requirements with only a few loose metal housings, but otherwise no relining or occlusal adjustments were necessary during a three-year follow-up period. The increased number of complications in our study compared to the other studies could be different types of attachment used or the longer follow-up period. This is supported by a systematic review and meta-analysis on the attachments used in IODs, where Chaware et al^3^ reported that locator attachments required more frequent maintenance and repairs.

The strength of this study lies in its prospective longitudinal design, with a 60-month follow-up period, which allows a comprehensive evaluation of peri-implant parameters in immediately placed and loaded mandibular IODs at multiple time points. This in turn enables a broad understanding of the long-term outcomes of this treatment approach. In addition, standardised protocols were used, which ensured that peri-implant parameters were measured and evaluated consistently across participants, enhancing the reliability of the study. Moreover, prosthetic complication frequency among IODs was also observed, which highlights the importance of evaluating a treatment protocol not only for its biological success but also for its clinical effectiveness. It is worth noting that previous research has provided limited information on prosthodontic maintenance in this context.

The present study focused on specific peri-implant parameters and prosthodontic maintenance, while other relevant factors such as Implant Stability Quotient (ISQ), the thickness of keratinised mucosa and patient-reported outcomes, e.g., patient satisfaction and quality of life, were not evaluated. It is also important to acknowledge that various confounding factors, including smoking habits, oral hygiene maintenance, and anatomical variations, might have potentially influenced the observed peri-implant parameters. However, this study contributes to the knowledge and understanding of peri-implant parameters in immediately placed and loaded mandibular overdentures, supporting the use of this treatment approach and guiding clinicians in providing optimal care to edentulous patients. Future research should consider expanding the longitudinal design with a longer follow-up period to assess the long-term durability and stability of peri-implant parameters. Increasing the sample size and incorporating assessments such as ISQ, keratinised mucosa thickness, patient satisfaction, and quality of life will provide a comprehensive understanding of immediate IOD treatment.

## CONCLUSION

Immediately placed and loaded mandibular implant overdentures using two non-splinted implants and locator attachments showed acceptable peri-implant parameters and functional outcome, including PI, BI and PIPD, over a 5-year follow-up period. This longer follow-up duration enabled us to identify changes in peri-implant parameters that occurred over time, thus shedding light on the treatment’s sustainability, durability and potential complications. A statistically significantly increase in marginal bone loss was observed from baseline to follow-up, but it remained within acceptable limits. A moderate number of restorative and abutment complications were observed during the follow-up of IODs. Immediately loaded IODs are an acceptable alternative treatment for edentulous mandibles, but adequate case selection and rigorous maintenance are critical for its good prognosis. The results of this study can provide valuable insights for dental professionals, guiding them in making evidence-based decisions and ultimately improving patient outcomes for edentulous patients.

## References

[ref1] Al­Rafee MA (2020). The epidemiology of edentulism and the associated factors: A literature review. J Family Med Prim Care.

[ref2] Attard NJ, Zarb GA (2005). Immediate and early implant loading protocols: a literature review of clinical studies. J Prosthet Dent.

[ref3] Chaware SH, Thakkar ST (2020). A systematic review and meta­analysis of the attachments used in implant­supported overdentures. J Indian Prosthodont Soc.

[ref4] Chen ST, Buser D, Sculean A, Belser UC (2023). Complications and treatment errors in implant positioning in the aesthetic zone: Diagnosis and possible solutions. Periodontol 2000.

[ref5] Duong HY, Roccuzzo A, Stähli A, Salvi GE, Lang NP, Sculean A (2022). Oral health­related quality of life of patients rehabilitated with fixed and removable implant­supported dental prostheses. Periodontol 2000.

[ref6] Elsyad MA, Al­mahdy YF, Fouad MM (2012). Marginal bone loss adjacent to conventional and immediate loaded two implants supporting a ball­retained mandibular overdenture: A 3­year randomized clinical trial. Clin Oral Implants Res.

[ref7] Elsyad MA, Mahanna FF, Elshahat MA, Elshoukouki AH (2016). Locators versus magnetic attachment effect on peri implant tissue health of immediate loaded two implants retaining a -mandibular overdenture: a 1 year randomised trial. J Oral Rehabil.

[ref8] Feine JS, Carlsson GE, Awad MA (2003). The McGill consensus statement on overdentures. Quintessence Int.

[ref9] Feine JS, Carlsson GE, Awad MA, Chehade A, Duncan WJ, Gizani S (2002). The McGill consensus statement on overdentures. Mandibular two­implant overdentures as first choice standard of care for edentulous patients. Int J Oral Maxillofac Implants.

[ref10] Gadallah AA, Youssef HG, Shawky YM (2012). A comparative study between early occlusal loading at 1 and 6?weeks in implant­retained mandibular overdentures. Implant Dent.

[ref11] Gowd MS, Shankar T, Ranjan R, Singh A (2017). Prosthetic consideration in implant­supported prosthesis: a review of literature. J Int Soc Prev Community Dent.

[ref12] Heckmann SM, Winter W, Meyer M, Weber HP, Wichmann MG (2001). Overdenture attachment selection and the loading of implant and denture­bearing area. Part 1: In vivo verification of stereolithographic model. Clin Oral Implants Res.

[ref13] Kronstrom M, Davis B, Loney R, Gerrow J, Hollender L (2014). A prospective randomized study on the immediate loading of mandibular overdentures supported by one or two implants; a 3?year follow­up report. Clin Implant Dent Relat Res.

[ref14] Kutkut A, Bertoli E, Frazer R, Pinto­sinai G, Fuentealba Hidalgo R, Studts JA (2018). A systematic review of studies comparing conventional complete denture and implant retained overdenture. J Prosthodont Res.

[ref15] Kutkut A, Rezk M, Zephyr D, Dawson D, Frazer R, Al­sabbagh M (2019). Immediate loading of unsplinted implant retained mandibular overdenture: a randomized controlled clinical study. J Oral Implantol.

[ref16] Mahoorkar S, Bhat S, Kant R (2016). Single implant supported mandibular overdenture: A literature review. J Indian Prosthodont Soc.

[ref17] Marzola R, Scotti R, Fazi G, Schincaglia G P (2007). Immediate loading of two implants supporting a ball attachment­retained mandibular overdenture: A prospective clinical study. Clin Implant Dent Relat Res.

[ref18] Misch CE (2014). Dental Implant Prosthetics.

[ref19] Misch CE, Wang HL, Misch CM, Sharawy M, Lemons J, Judy KW (2004). Rationale for the application of immediate load in implant dentistry: part II. Implant Dent.

[ref20] Mombelli A, van Oosten MA, Schurch Ejr, Land NP (1987). The microbiota associated with successful or failing osseointegrated titanium implants. Oral Microbiol Immunol.

[ref21] Payne AG, Alsabeeha NH, Atieh MA, Esposito M, Ma S, Anas El­wegoud M (2018). Interventions for replacing missing teeth: attachment systems for implant overdentures in edentulous jaws. Cochrane Database Syst Rev.

[ref22] Qureshi AW, Rahim S, Abbasi MS, Akhtar Q, Qureshi SW (2019). Oral stereognostic score in edentulous patients. Pakistan Oral Dent J.

[ref23] Raustia AM, Salonen MA, Pyhtinen J (1996). Evaluation of masticatory muscles of edentulous patients by computed tomography and electromyography. J Oral Rehabil.

[ref24] Renouard F, Renouard E, Rendón A, Pinsky HM (2000). Increasing the margin of patient safety for periodontal and implant treatments: The role of human factors. Periodontol.

[ref25] Roccuzzo A, Imber JC, Salvi GE, Roccuzzo M (2000). Peri­implantitis as the consequence of errors in implant therapy. Periodontol.

[ref26] Salman A, Thacker S, Rubin S, Dhingra A, Ioannidou E, Schincaglia GP (2019). Immediate versus delayed loading of mandibular implant retained overdentures: a 60 month follow up of a ---randomized clinical trial. J Clin Periodontol.

[ref27] Schimmel M, Srinivasan M, Herrmann FR, Müller F (2014). Loading protocols for implant­supported overdentures in the edentulous jaw: a systematic review and meta­analysis. Int J Oral Maxillofac Implants.

[ref28] Seo YH, Bae EB, Kim JW, Lee SH, Yun MJ, Jeong CM, Jeon YC, Huh JB (2016). Clinical evaluation of mandibular implant overdentures via Locator implant attachment and Locator bar attachment. J Adv Prosthodont.

[ref29] Sharka R, Abed H, Hector M (2019). Oral health­related quality of life and satisfaction of edentulous patients using conventional complete dentures and implant­retained overdentures: An umbrella systematic review. Gerodontol.

[ref30] Stephan G, Vidot F, Noharet R, Mariani P (2007). Implant­retained mandibular overdentures: a comparative pilot study of immediate loading versus delayed loading after two years. J Prosthet Dent.

[ref31] Stoker GT, Wismeijer D (2011). Immediate loading of two implants with a mandibular implant­retained overdenture: A new treatment protocol. Clin Implant Dent Relat Res.

[ref32] Stricker A, Gutwald R, Schmelzeisen R, Gellrich NG (2004). Immediate loading of 2 interforaminal dental implants supporting an overdenture: clinical and radiographic results after 24months. Int J Oral Maxillofac Implants.

[ref33] Turkyilmaz I, Tumer C, Avci M, Hersek N, Celik­bagci E (2006). A short­term clinical trial on selected outcomes for immediately loaded implant­supported mandibular overdentures. Int J Prosthodont.

[ref34] Vercruyssen M, Marcelis K, Coucke W, Naert I, Quirynen M (2010). Long­term, retrospective evaluation (implant and patient­centred outcome) of the two­implants­supported overdenture in the mandible. Part 1: survival rate. Clin Oral Implants Res.

[ref35] Zygogiannis K, Aartman IH, Parsa A, Tahmaseb A, Wismeijer D (2017). Implant mandibular overdentures retained by immediately loaded implants: a 1­year randomized trial comparing the clinical and radiographic outcomes between mini dental implants and standard­sized implants. Int J Oral Maxillofac Implants.

[ref36] Zygogiannis K, Wismeijer D, Aartman IH, Osman RB (2016). A systematic review on immediate loading of implants used to support overdentures opposed by conventional prostheses: factors that might influence clinical outcomes. Int J Oral Maxillofac Implants.

